# The Aromatic Head Group of Spider Toxin Polyamines Influences Toxicity to Cancer Cells

**DOI:** 10.3390/toxins9110346

**Published:** 2017-10-27

**Authors:** David Wilson, Glen M. Boyle, Lachlan McIntyre, Matthew J. Nolan, Peter G. Parsons, Jennifer J. Smith, Leon Tribolet, Alex Loukas, Michael J. Liddell, Lachlan D. Rash, Norelle L. Daly

**Affiliations:** 1 Centre for Biodiscovery and Molecular Development of Therapeutics, AITHM, James Cook University, Cairns, QLD 4878, Australia; david.wilson4@jcu.edu.au (D.W.); mjnolan78@gmail.com (M.J.N.); leon.tribolet@gmail.com (L.T.); alex.loukas@jcu.edu.au (A.L.); 2 QIMR Berghofer Medical Research Institute, Herston, QLD 4006, Australia; Glen.Boyle@qimrberghofer.edu.au (G.M.B.); Peter.Parsons@qimr.edu.au (P.G.P.); 3 Centre for Tropical Environmental and Sustainable Sciences, James Cook University, Cairns, QLD 4878, Australia; lach.mcintyre@gmail.com (L.M.); michael.liddell@jcu.edu.au (M.J.L.); 4 Institute for Molecular Bioscience, The University of Queensland, Brisbane, QLD 4072, Australia; jennifer.smith@imb.uq.edu.au (J.J.S.); l.rash@uq.edu.au (L.D.R.); 5 School of Biomedical Sciences, University of Queensland, Brisbane, QLD 4072, Australia

**Keywords:** spider venom, NMR spectroscopy, polyamine, cancer, cytotoxicity

## Abstract

Spider venoms constitute incredibly diverse libraries of compounds, many of which are involved in prey capture and defence. Polyamines are often prevalent in the venom and target ionotropic glutamate receptors. Here we show that a novel spider polyamine, PA_366_, containing a hydroxyphenyl-based structure is present in the venom of several species of tarantula, and has selective toxicity against MCF-7 breast cancer cells. By contrast, a polyamine from an Australian funnel-web spider venom, which contains an identical polyamine tail to PA_366_ but an indole-based head-group, is only cytotoxic at high concentrations. Our results suggest that the ring structure plays a role in the cytotoxicity and that modification to the polyamine head group might lead to more potent and selective compounds with potential as novel cancer treatments.

## 1. Introduction

The discovery of new compounds from nature is still one of the most efficient methods for finding lead molecules for the development of pharmaceuticals [1,2]. These new compounds range from small molecules to large biologics, and several approaches have facilitated their discovery. Screening using NMR spectroscopy and recent advances in metabolomics have been valuable for characterising novel small molecules, whereas advances in genomics and proteomics, as well as high-throughput screening approaches are providing novel methods for the discovery of peptides and proteins [3,4].

In nature, venomous creatures are a rich source of new bioactive compounds. In particular, spider venoms have enormous compound diversity, much of which has yet to be explored. More than four million compounds are estimated to be present in the venom of the 46,000 different spider species [5,6]. Spider venom components are present for prey capture and defence purposes; however, some venom compounds have been shown to have therapeutic potential, such as in the treatment of pain and inflammation [7]. While the majority of compounds present in spider venoms are disulfide-rich peptides [5], small molecules such as the sulfated nucleosides found in brown recluse spiders [8] and polyamines, found in many spider species (reviewed in [9]), are also present.

Polyamines are cationic compounds widespread throughout nature and are found in both prokaryotic and eukaryotic organisms. They can modulate a range of cellular processes that include cell proliferation, signalling and ion channel function [10]. The biological activities of polyamines are mediated through interaction with anionic molecules including DNA, RNA and proteins. However, such structural diversity exists amongst the polyamines that some have been shown to be involved in tumorigenesis [11], and some have shown potential in treating diseases such as cancer [12]. Polyamines with potential for treating diseases include examples from marine sponges and fungi that inhibit carbonic anhydrase IX, a cancer drug target [13]. One study that showed the conjugation of certain polyamines to chloramphenicol enhanced anti-cancer and anti-bacterial activity [14]. The polyamine toxins present in spider venoms are part of a sub-class of polyamines characterised by an aromatic head group with a polyamine chain that can block ionotropic glutamate (iGlu) receptors [12,15]. These receptors have been considered as promising drug targets for neurological and psychiatric disorders [16].

Using a combination of NMR and bioassay screening, we have identified and characterised a novel polyamine (PA_366_) present in a range of tarantula (Theraphosidae) venoms. PA_366_ contains a hydroxyphenyl-based aromatic head group and has more potent cytotoxicity against MCF-7 breast cancer cells than SK-MEL-28 melanoma or neonatal foreskin fibroblast (NFF) primary cells. Potential protein binding interactions were probed using protein array analysis to investigate the mechanism of action, and a comparison with an indole-based polyamine from Australian funnel-web spider venom provided insight into the structure/function relationships.

## 2. Results

### 2.1. Cytotoxicity Assays and Characterisation of Bioactive Compound

**Figure 1:**
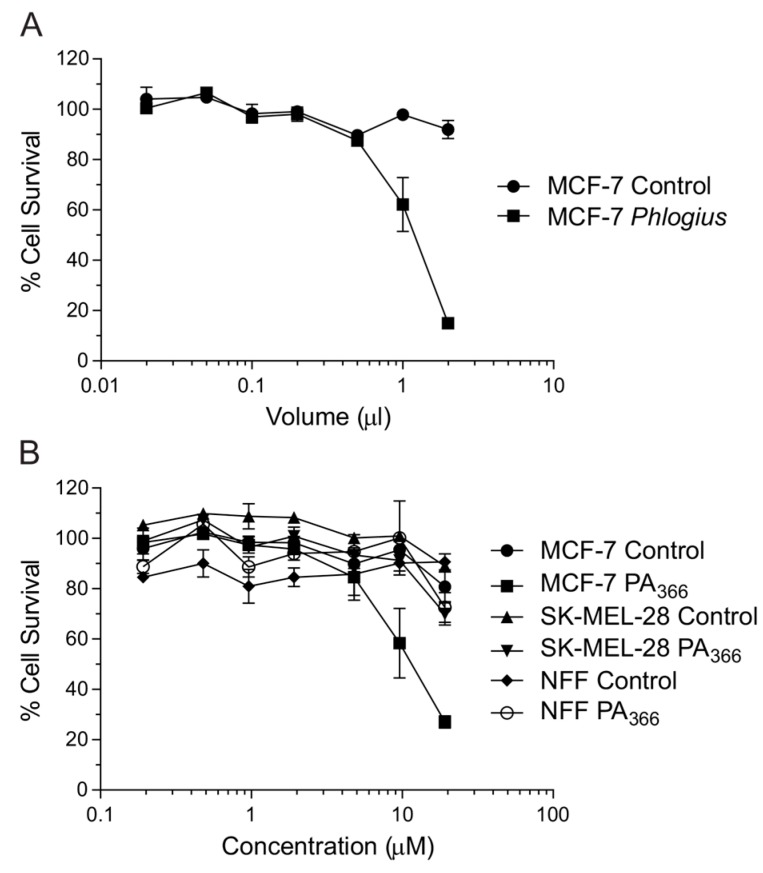
Cytotoxicity of *Phlogius* sp. crude venom and a purified component, PA_366_. (**A**) Dose response for cytotoxic cell killing by *Phlogius* sp. crude venom compared to vehicle. Crude venom (squares) or vehicle (circles) was incubated with MCF-7 breast cancer cells at the indicated volumes for 5 days, before assay for cell survival using the sulforhodamine B (SRB) assay. Cell survival percentages normalised to untreated cells are indicated. (**B**) Dose response for cytotoxic cell death of PA_366_ compared to vehicle in MCF-7, SK-MEL-28 or NFF cells. Cells were treated with the indicated concentrations of purified PA_366_ for 5 days, before assay for cell survival using SRB. Cell survival percentages normalised to untreated cells are indicated. Representative data from a single experiment with triplicate readings are shown.

Crude venoms from female specimens of 31 species of tarantula (Theraphosidae) were screened for activity against the MCF-7 breast cancer cell line. Crude venom from funnel web spiders (male Sydney Funnel-web spider (*Atrax robustus*), female *Hadronyche valida*, *Hadronyche cerberea* and *Hadronyche infensa- including species variants*), and the wolf spider *Hogna carolinensis*, was also tested to allow comparison with venom from two other families (Hexathelidae and Lycosidae). Substantial cytotoxicity activity (defined as greater than 50% decrease in absorbance relative to the negative control) was observed for 17 of the species tested including examples from all three families tested (see Table S1 for the complete list of species screened). The cytotoxicity observed with crude female *Phlogius* sp. spider venom is shown in Figure 1A.

Venom from eight species that displayed cytotoxicity were chosen for further study based on availability of sample, and are listed in Table 1. Crude venom from these eight species were fractionated using reversed-phase high performance liquid chromatography (RP-HPLC), and one-minute fractions were collected. The fractions were subsequently tested against two cancer cell lines, MCF-7 and SK-MEL-28 cells (melanoma cell line) in a clonogenic-type assay. These cell lines were chosen to cover different origins of cancer as MCF-7 are epithelial based and the SK-MEL-28 cells are from neural crest. NFF cells were also tested to represent normal cells with a doubling time similar to cancer cell lines. Variation was observed in activity between the fractionated venoms, and is consistent with venom component variations between spiders as shown previously for related species [5,17]. Despite this variation, all eight species showed early eluting fractions in the RP-HPLC chromatograms that demonstrated activity against the MCF-7 breast cancer cell line.

Due to the availability of suitable quantities of the *Phlogius* sp. spider venom, this species was chosen for larger scale purification and characterisation of the active component(s). The active fractions from the *Phlogius* sp. spider venom were analysed using mass spectrometry and analytical RP-HPLC, and shown to contain a single major component. This purified component, termed PA_366_, was tested for activity in the clonogenic-type assays. PA_366_ showed activity against MCF-7 cells, and limited activity against the SK-MEL-28 and primary NFF cells when compared to vehicle controls as shown in Figure 1B.

**Figure 2:**
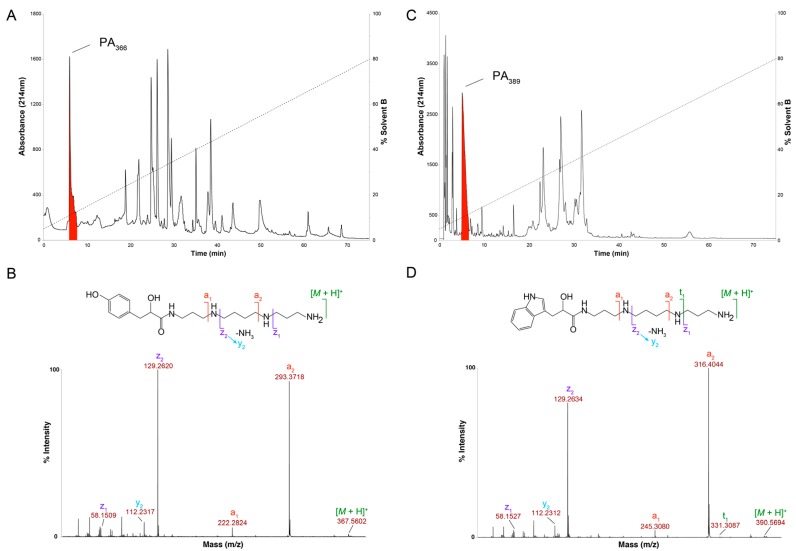
Characterisation of spider venom polyamines. (**A**) RP-HPLC chromatogram of crude female *Phlogius* sp. venom with the peak corresponding to the polyamine PA_366_ highlighted in red (Thermo Scientific Hypersil GOLD aQ 250 × 10 mm, 5 µm column; 1 mL/min flow rate; Solvent A H_2_O/0.05% TFA, Solvent B 90% ACN/H_2_O/0.045% TFA; 5–80% solvent B in 75 min, 80–90% solvent B in 5 min, 90% solvent B for 5 min, and 90–5% solvent B in 2 min; absorbance at 214 nm); (**B**) SCIEX TOF/TOF™ 5800 MALDI MS/MS spectrum of PA_366_ using CHCA matrix, and the determined chemical structure with relevant fragment ions highlighted; (**C**) RP-HPLC chromatogram of crude female *A. robustus* venom with the peak corresponding to the polyamine PA_389_ highlighted in red (Thermo Scientific Hypersil GOLD aQ 250 × 10 mm, 5 µm column; 1 mL/min flow rate; Solvent A H_2_O/0.05% TFA, Solvent B 90% ACN/H_2_O/0.045% TFA; 5–80% solvent B in 75 min, 80–90% solvent B in 5 min, 90% solvent B for 5 min, and 90–5% solvent B in 2 min; absorbance at 214 nm); (**D**) SCIEX TOF/TOF™ 5800 MALDI-MS/MS spectrum of PA_389_ using CHCA matrix, and the determined chemical structure with relevant fragment ions highlighted.

**Figure 3:**
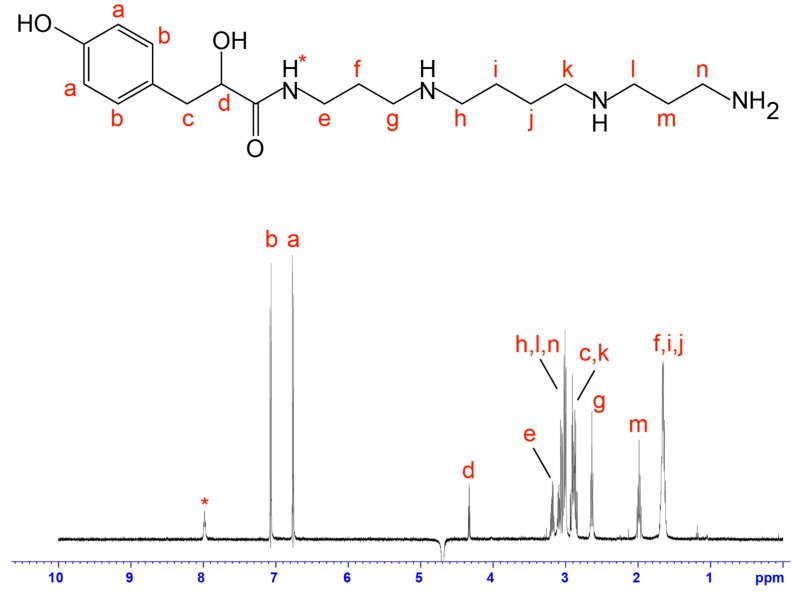
Chemical structure and ^1^H NMR spectrum of PA_366_. The assignments were derived based on two-dimensional NMR spectra and confirmed using mass spectrometry fragmentation analysis.

PA_366_ is highly hydrophilic and is one of the first peaks of crude *Phlogius* sp. spider venom to elute from a C_18_ RP-HPLC column (Figure 2A). Analysis of the homonuclear and heteronuclear NMR data of PA_366_ indicated the presence of a ring structure connected to a spermine polyamine tail, as shown in Figure 2B. This structure was elucidated based on correlations observed in the HSQC, HMBC and HSQC-TOCSY spectra, and coupling constants and chemical shifts measured from one-dimensional ^1^H spectra. The multiplicities of the ^1^H NMR signals are reported as: d, doublet; t, triplet; p, pentet; m, multiplet; br, broad. Coupling constants (*J* values) are reported in hertz (Hz). HRMS: calculated for C_19_H_34_N_4_O_3_ [M + H]^+^ 367.2631, found 367.2653. ^1^H NMR (600 MHz, H_2_O/10% D_2_O): δ 8.06 (1H, br t, *J* = 6.21 Hz, H_*_), 7.16 (2H, d, *J* = 8.71 Hz, H_b_), 6.85 (2H, d, *J* = 8.71 Hz, H_a_), 4.41 (1H, t, *J* = 5.96 Hz, H_d_), 3.31–3.25 (2H, m, H_e_), 3.20–3.08 (6H, m, H_n_, H_h_, H_l_), 3.02–2.93 (4H, m, H_c_, H_k_), 2.73 (2H, br t, *J* = 8.72 Hz, H_g_), 2.08 (2H, br p, H_m_), 1.78–1.71 (6H, br m, H_i_, H_j_, H_f_). ^13^C NMR (150 MHz, H_2_O/10% D_2_O): δ 179.0, 156.7, 133.3 (2C), 130.7, 118.3 (2C), 73.7, 48.8, 48.6, 46.7, 46.2, 40.4, 38.4, 37.4, 27.1, 25.5, 24.3 (2C). The proton assignments are displayed on the one-dimensional spectrum in Figure 3. MS/MS fragmentation resulted in two primary fragment ions of *m*/*z* 129.26 and *m*/*z* 293.37, consistent with fragmentation at the sites labelled in Figure 2B. Other fragment ions were also evident including *m*/*z* 58.15, *m*/*z* 112.23 and *m*/*z* 222.28, and are consistent with the fragmentation patterns observed in similar molecules [18]. The theoretical calculated mass for the derived structure of PA_366_ is 366.2631 Da, and a *m*/*z* 367.2653 (exact mass 366.2573 Da) was observed by MALDI-MS in reflector positive ion mode.

### 2.2. NMR Screening of Crude Spider Venoms

**Figure 4:**
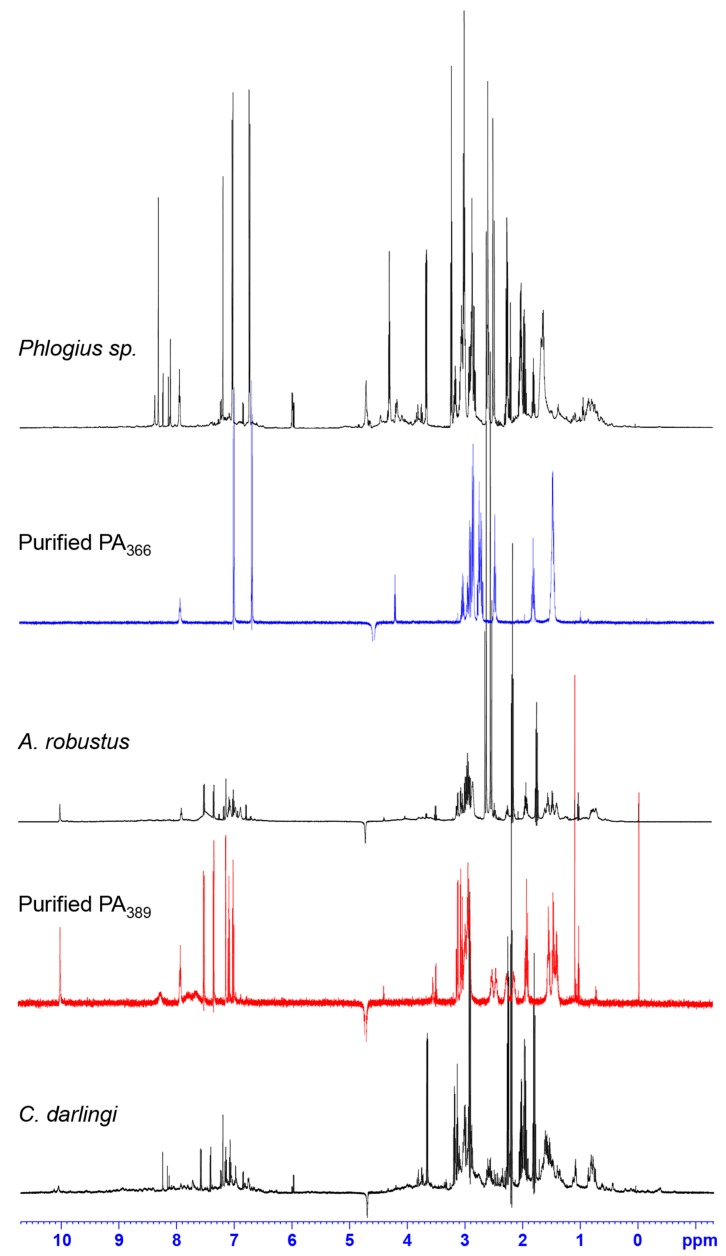
One-dimensional NMR spectra of selected crude spider venoms and purified polyamines. ^1^H NMR spectra of crude venom from *Phlogius* sp., *A. robustus* and *C. darlingi* recorded at 600 MHz, showing the presence of PA_366_ in the *Phlogius* sp. venom, and PA_389_ in the *A. robustus* and *C. darlingi* venom. The ^1^H NMR spectra of purified PA_366_ and PA_389_ recorded at 600 MHz are also shown.

Following the characterisation of purified PA_366_, crude venom from the seven other spiders that showed early eluting fractions with cytotoxic activity were analysed using one-dimensional NMR spectroscopy to screen for the presence of PA_366_. The one-dimensional NMR spectra of selected examples are shown in Figure 4. Although spider venoms have numerous disulfide-rich peptides, the small molecules have sharper peaks in the 1D spectra, making them easily discernible from peptide signals. This analysis confirmed the presence of PA_366_ in the crude venom from the spider species *Acanthoscuria geniculata, Chilobrachys penang, and Psalmopoeus irminia,* in addition to the *Phlogius* sp.

**Figure 5:**
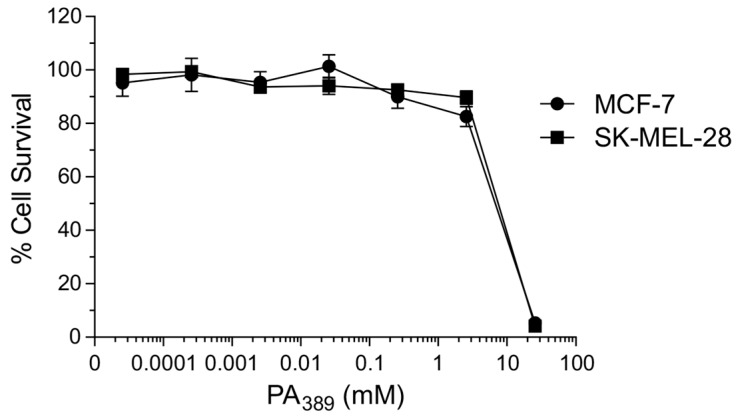
Cytotoxicity of PA_389_ against cancer cell lines. Dose response for cytotoxic cell killing of PA_389_ in MCF-7 (circles) or SK-MEL-28 (squares) cells. Cells were treated with the indicated concentrations of purified PA_389_ for five days, before assay for cell survival was assessed using SRB. Cell survival percentages normalised to untreated cells are indicated. Representative data from a single experiment with triplicate readings are shown.

Analysis of the NMR spectra of the crude venom of *Ceratogyrus darlingi* and *A*. *robustus* indicated they do not contain PA_366_, despite demonstrating cytotoxic activity in the assays. The lack of PA_366_ was confirmed by mass spectrometry analysis, and instead a mass of 389 Da was present. The structure of this active component in *A. robustus* venom was determined following further purification by RP-HPLC, and subsequent analysis using NMR spectroscopy and MS/MS. The analysis determined that *A. robustus* venom contains a polyamine previously characterised from the venom of a trap-door spider (*Hebestatis theveniti*) and a tarantula (*Harpactirella* sp.), and termed Het_389_ [19]. Subsequent analysis of the *C. darlingi* venom also confirmed the presence of Het_389_. Given the presence of Het_389_ across multiple genera, we suggest the new identifying term PA_389_ is more appropriate. The structure of PA_389_ is shown in Figure 2D and the 1D NMR spectra in Figure 4. The theoretical calculated exact mass for PA_389_ is 389.2791 Da, and a *m*/*z* 390.2443 (exact mass 389.2364 Da) was observed by MALDI-MS in reflector positive ion mode. In comparison to the hydroxyphenyl based head group in PA_366_, PA_389_ has an indole head group and identical spermine tail. The cytotoxicity of PA_389_ is shown in Figure 5 and shows this polyamine only displays cytotoxicity at millimolar concentrations.

**Figure 6:**
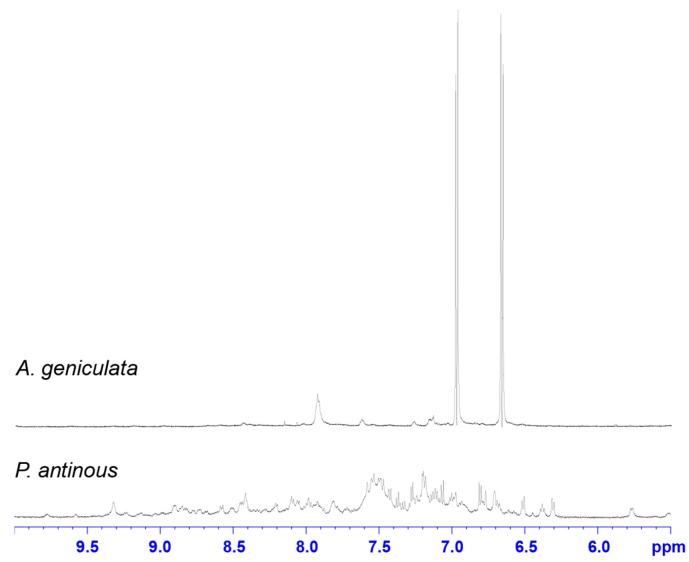
One-dimensional NMR spectra of selected crude spider venoms from *P. antinous* and *A. geniculata*. ^1^H NMR analysis of the crude venom from *P. antinous* shows a composition that is primarily peptides, based on the number of peaks and dispersion in the amide region; this venom does not show any evidence of cytotoxicity against MCF-7, SK-MEL-28 or NFF cells. In contrast, the spectrum of cytotoxic crude venom from *A. geniculata* is dominated by the peaks corresponding to PA_366_.

The venom from *H. gigas* and *N. chromatus* did not appear to contain either PA_366_ or PA_389_, despite displaying cytotoxic activity. *H. gigas* appears to contain primarily peptide based molecules, whereas the spectra from *N. chromatus* is dominated by small molecules but with different shifts to the polyamines characterised in this study. Comparison of the 1D spectra of *A. geniculata* and *Pamphobeteus antinous*, a tarantula species that did not display cytotoxic activity, highlights the differences in the venom NMR “profiles” in the presence and absence of small molecules, as shown in Figure 6. The NMR spectra of *A. geniculata* is dominated by PA_366_, whereas *P. antinous* shows no evidence of small molecules. Instead, the venom of *P. antinous* appears to contain primarily peptides based on the large number of peaks and significant peak dispersion in the 6–9.5 ppm range. These peptides are likely to be disulfide-rich based on previous analyses of spider venoms [20].

### 2.3. Protein Array Analysis

A sample of PA_366_ was biotinylated for protein array analysis to identify any protein binding interactions. Biotinylated PA_366_ was shown by MS analysis to have a molecular weight of 706 Da, confirming biotin binding to the primary amine at the end of the polyamine chain. Analysis of the biotinylated PA_366_ binding to a human protein array demonstrated interaction with numerous proteins, presumably via non-specific interactions. Signals with Z-scores above three were considered to be significant. The polyamine bound to MAPK1 and MAP3K2; both giving Z-scores of 3.7. These proteins have previously been shown to have roles in tumour development and breast cancer [21]. The protein with the highest score however, based on the signal intensity, was a zinc finger protein (ZNF501 Z-score 13.93).

## 3. Discussion

In the current study, we have used NMR spectroscopy and MS to characterise the novel polyamine PA_366_, which has selective cytotoxicity for MCF-7 breast cancer cells. Comparison with a related polyamine, PA_389_, provided insight into the important structural features. The two polyamines consist of a spermine tail functionalised with an aromatic head group, and are identical except PA_366_ has a 2-hydroxy-3-(4-hydroxyphenyl)propanal aromatic head group, while PA_389_ contains a 2-hydroxy-3-(1*H*-indol-3-yl)propanal based head group. PA_389_ only displays cytotoxicity at high concentrations in contrast to PA_366_, indicating that the aromatic head group is important for cytotoxicity.

Using ProtoArray technology, analysis of the protein binding properties of PA_366_ indicates non-specific binding to a range of proteins. However, the protein with the highest Z-score in the protein array analysis, which is likely to have the most significant interaction, is zinc finger protein 501, which belongs to the Krüppel-like c2h2 type zinc finger protein family. Zinc finger proteins are one of the most common families of DNA-binding transcription factors [22] and a recent study suggested they can be involved in tumour development [21]. Interestingly, the mammalian polyamines putrescine, spermidine and spermine alter binding of DNA to zinc finger transcription factors [23], which is consistent with a role for zinc finger protein 501 in the bioactivity of PA_366_. However, additional interactions might also be involved given that MAPK1 and MAP3K2, mitogen-activated protein kinases (MAPK), were also significant hits in the ProtoArray analysis. Several MAPK genes are associated with breast cancer [24], and activation of MAPK can lead to cell proliferation, invasion and metastasis, making these kinases potential targets for cancer treatment [25,26]. To provide greater insight into the possible targets of PA_366_ it is important to determine the membrane permeability, which will influence the targets accessible to the molecule.

The cytotoxicity we observed for PA_366_ is consistent with the role of mammalian polyamines in cell death. Numerous links have been identified between mammalian polyamines and apoptotic pathways. For example, the production of hydrogen peroxide produced during polyamine catabolism is thought to be involved in cell death [27]. However, alteration in polyamine levels can have an impact on numerous cellular functions such as DNA-protein interactions, protein-protein interactions and mitochondrial integrity which can lead to cell death. Morphological analysis of the cells following incubation with PA_366_ indicates the presence of apoptosis but further study is required to confirm this hypothesis.

Although PA_366_ displays cytotoxicity against a cancer cell lines, this is unlikely to be related to its function in the spider venom. The primary target of venom polyamine toxins is thought to be the ionotropic glutamate receptors [28]. PA_389_ has previously been shown to cause rapid but reversible paralysis to lepidopteran insect larvae, suggesting a role in prey capture [19]. Given the structural and functional similarities between PA_389_ and PA_366_, it is likely that the latter also displays some form of bioactivity against insect prey.

**Figure 7:**
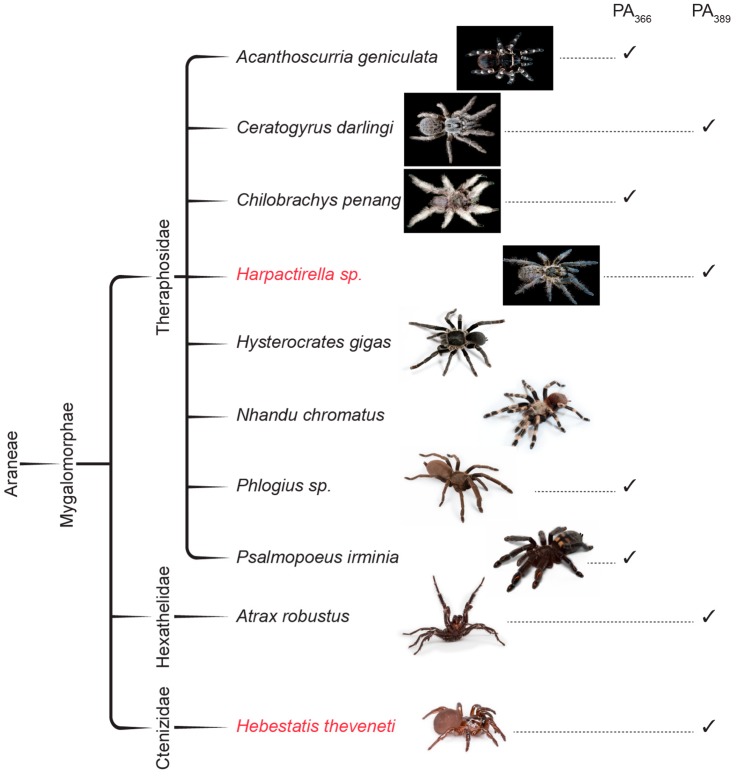
Phylogenic tree of some spider venoms showing the distribution of PA_366_ and PA_389_. A phylogenetic tree of crude spider venoms that demonstrated cytotoxicity on MCF-7 or SK-MEL-28 cells in this study, and the presence of the polyamines PA_366_ and PA_389_. The two spider species, *Hebestatis theveneti* and *Harpactirella* sp., from the original identification and characterisation study of PA_389_ are also included [19] (Photograph credits: *Acanthoscurria geniculata*, *Ceratogyrus darlingi*, *Harpactirella* sp., *Hysterocrates gigas*, and *Nhandu chromatus*—Bastian Rast; *Chilobrachys penang*—Muhammad Ashraf; *Psalmopoeus irminia*—Edward Evans; *Hebestatis theveneti*—Marshal Hedin; *Phlogius* sp. and *Atrax robustus*—David Wilson).

The known distribution of PA_366_ and PA_389_ determined from previous work and this study is shown in the phylogenetic tree in Figure 7. PA_366_ is present in a range of geographically diverse spider species from the family Theraphosidae, indicating that differences such as diet and environmental conditions are unlikely to influence the production of this polyamine. Similarly, PA_389_ is present in the venom of spiders from the Theraphosidae family, but also in the venom of species from the Hexathelidae and Ctenizidae families. In this study, we have confirmed the presence of PA_389_ in the venom of the Sydney Funnel-web spider, *Atrax robustus*, as the undefined 3-(3-indoyl)lactic acid and spermine complex suggested by Sutherland [29] and proposed as identical to PA_389_ by Skinner et al. [19]. It is likely that PA_366_ is also present in other spider families but further analysis is required to confirm this hypothesis.

One outcome of this study is NMR spectroscopy proved an effective way of screening and detecting the presence of the polyamines PA_366_ and PA_389_ without the need for fractionation of the venom. Although the spider venoms contain a large number of disulfide-rich peptides, the polyamines have low molecular weights and provide much sharper spectral peaks, and can be detected when only low microgram amounts of material are present in contrast to the larger peptides. This type of NMR spectroscopy screening has also been shown to be useful for the discovery of new small molecules from other sources. For example, unfractionated extracts from insects examined using 2D NMR spectroscopy led to the discovery of tricyclic pyrones from a ladybird beetle including the identification of ring systems that had not previously been found in nature [1].

Despite the conservation of PA_366_ in several venoms, our study also serves to highlight the compound diversity and broad range of biological activities found in spider venom. *N. chromatus* has early eluting peaks from RP-HPLC that display cytotoxicity but the venom does not appear to contain PA_366_ or PA_389_. The 1D NMR spectrum indicates the presence of small molecules, and it is likely there are several, related polyamines that might account for our observed activity. Although there is evidence of small molecules in the crude venom of *H. gigas*, the venom is dominated by peptides/proteins in contrast to the venoms such as *Phlogius* sp., *A. robustus* and *C darlingi*. Furthermore, *P. antinous* appears to show only peptide related peaks in the amide region, and does not display cytotoxicity. This compound and bioactivity diversity is one of the features that has led to the extensive study of spider venom for the discovery of novel compounds for therapeutic or agricultural applications [20].

In summary, we have shown that a spider venom polyamine can have relatively selective cytotoxicity activity against specific cancer cell lines and that the aromatic head group appears to be involved in conferring cytotoxicity. Insight into the distribution of spider venom polyamines across species and genera has also been gained from screening using NMR spectroscopy.

## 4. Materials and Methods

### 4.1. Venom Collection and Purification

A majority of the crude venoms were purchased from the commercial supplier Alphabiotoxine Laboratory, Montroeul-au-bois, Hainaut, Belgium. Crude venom from *Pterinochilus meridionalis*, *Vitalius roseus* and *Hogna carolinensis* was purchased from SpiderPharm, Yarnell, AZ, USA. Crude venom was also collected from a colony of housed specimens of female Australian tarantulas (*Phlogius* sp. and *Coremiocnemis tropix*), *Macrothele gigas*, and *Poecilotheria fasciata* and *Poecilotheria metallica* by electrostimulation of the venom glands. Male Sydney funnel-web spider (*Atrax robustus*) venom (~5 mg) was supplied by the Australian Reptile Park (Gosford, NSW, Australia). Crude venom was fractionated by reversed-phase HPLC (RP-HPLC) using an Agilent 1260 Infinity HPLC system (Agilent, Santa Clara, CA, USA) and a Thermo Scientific Hypersil GOLD aQ (250 × 10 mm, 5 µm) column (Thermo Fisher Scientific, Waltham, MA, USA). Fractionation of the venom components was achieved using a linear gradient of two mobile phases: H_2_O/0.05% trifluoroacetic acid (TFA; Auspep, Tullamarine, VIC, Australia) [solvent A], and 90% acetonitrile (ACN; Sigma-Aldrich, St. Louis, MO, USA)/H_2_O/0.045% TFA [solvent B]. Separation used a gradient of 5–80% solvent B in 75 min, 80–90% solvent B in 5 min, 90% solvent B for 5 min, and 90–5% solvent B in 2 min at a flow rate of 6 mL/min. Venom component elution was monitored at 214 nm and 1 min fractions were collected.

### 4.2. Cytotoxicity Assays

All cells were cultured in RPMI-1640 medium supplemented with 10% heat-inactivated foetal calf serum (Life Technologies, Carlsbad, CA, USA) in RPMI-1640 medium supplemented with 100 U/mL penicillin, 100 µg/mL streptomycin, and 3 mM HEPES at 5% CO_2_, 99% humidity at 37 °C. Routine mycoplasma tests were performed using Hoechst stain or PCR and were always negative. The cytotoxicity of compounds was determined using clonogenic survival assays of MCF-7 (breast adenocarcinoma), SK-MEL-28 (melanoma) and human neonatal foreskin fibroblasts (NFF) cells [30,31]. The cell lines were purchased from ATCC. Cells were plated into 96-well microtitre plates at 5 × 10^3^ cells/well, and allowed to adhere overnight. Compounds were added to culture medium at the indicated final concentrations, and plates incubated under the above conditions for five days. Cell survival was then assayed using sulforhodamine B (SRB; Sigma, St. Louis, MO, USA). Briefly, the culture medium was removed from the 96-well microtitre plates and the plates washed twice with phosphate buffered saline (PBS), before the cells were fixed with methylated spirits for 15 min. The plates were then rinsed with tap water and the fixed cells stained with 50 µL/well of SRB solution (0.4% sulforhodamine B (*w*/*v*) in 1% (*v*/*v*) acetic acid) over a period of 1 h. The SRB solution was removed from the wells and the plates rapidly washed two times with 1% (*v*/*v*) acetic acid. Protein bound dye was then solubilised with the addition of 100 µL of 10 mM unbuffered Tris, and incubated for 15 min at 25 °C. Plates were then read at 564 nm on a VERSA max tuneable microplate reader (Molecular Devices, Sunnyvale, CA, USA). Negative (vehicle only) and positive (cisplatin) controls were run with every cell survival experiment to ensure the quality of the data. Vehicle only control results in <5% change in cell survival whereas the positive control inhibited cell survival at nanomolar concentrations as expected.

### 4.3. Mass Spectrometry and NMR Analysis

^1^H NMR and ^13^C NMR spectra were recorded at 600.13 MHz and 150.91 MHz at 298 K on a Bruker Avance III 600 MHz spectrometer (Bruker, Billerica, MA, USA) equipped with a cryoprobe. Samples were dissolved in 90% H_2_O/10% D_2_O (*v*/*v*) (100 μM). D_2_O (99.9%) was obtained from Cambridge Isotope Laboratories, Woburn, MA, USA for ^1^H NMR measurements. Spectra were referenced to 4,4-dimethyl-4-silapentane-1-sulfonic acid (DSS; Cambridge Isotope Laboratories, Woburn, MA, USA). Two-dimensional spectra included TOCSY, NOESY, DQF-COSY, HSQC, HMBC and HSQC-TOCSY. TOCSY and NOESY mixing times of 80 ms and 500 ms respectively were used. Spectra were analyzed using TOPSPIN (Bruker, Billerica, MA, USA).

Mass spectrometry (MS) was performed using a SCIEX TOF/TOF™ 5800 MALDI, and high resolution mass spectrometry (HRMS) using direct infusion on a SCIEX QSTAR Elite equipped with a nanospray (SCIEX, Framingham, MA, USA). MALDI-MS samples were spotted on 384-well stainless steel target plates using 0.5 μL of sample and 0.5 μL of either *α*-cyano-4-hydroxycinnamic acid (CHCA; Sigma-Aldrich, St. Louis, MO, USA) matrix at 7.5 mg/mL in 50% ACN/0.1% TFA, or 2,5-dihydroxybenzoic acid (DHB; Sigma-Aldrich, St. Louis, MO, USA) matrix at 10 mg/mL in 50% ethanol/0.1% TFA. Calibration was performed before spectra collection for each sample using Calibration Mix solution 2 (SCIEX, Framingham, MA, USA). Spectra were acquired in reflector positive ion mode from *m*/*z* 300 to 1000 Da, and averaged over 2000 laser shots. QSTAR Elite MS samples were infused at 2 µL/min. MS spectra were acquired in positive ion mode from *m*/*z* 200 to 1000 Da and spectra were externally calibrated using SCIEX renin standard. MS/MS spectra were acquired for the product ions 367.26 Da and 390.2 Da from *m*/*z* 10 to 500 Da. All spectra were acquired with an accumulation time of 1 s, using an ion-spray voltage of 3700 V, a declustering potential of 80 V, and a focussing potential of 280 V.

### 4.4. Protein Array Analysis

Biotinylation of PA_366_ for protein interaction analysis was achieved utilising a 20-fold excess of EZ-Link^®^ Sulfo-NHS-LC-biotin (ThermoFisher Scientific, Waltham, MA, USA), and following the manufacturer’s instructions. Successful labelling of PA_366_ was confirmed by molecular weight analysis using a SCIEX TOF/TOF™ 5800 mass spectrometer (SCIEX, Framingham, MA, USA), followed by RP-HPLC purification of labelled PA_366_ to remove excess non-reacted and hydrolyzed biotin reagent from the solution. The purified sample was dried and resuspended in 100 µL of 1.0% PBS (pH 7.2) to achieve a required final amount of 10 µg. Interactions between biotinylated PA_366_ and more than 9000 human proteins were investigated employing a ProtoArray^®^ Human Protein Microarray v5.0 Protein-Protein Interaction (PPI) kit (Invitrogen™, Carlsbad, CA, USA), and significant interactions detected using a GenePix^®^ 4000Bmicroarray scanner (Molecular Devices, Sunnyvale, CA, USA), according to the manufacturer’s instructions.

## Figures and Tables

**Table 1:** Spider venoms with cytotoxic activity and the presence or absence of two polyamines ^a^.

Species	PA_366_	PA_389_
*Acanthoscuria geniculata*	×	
*Ceratogyrus darlingi*		×
*Chilobrachy penang*	×	
*Histerocrastes gigas*		
*Nhandu chromatus*		
*Phlogius* sp.	×	
*Psalmopoeus irminia*	×	
*Atrax robustus*		×

^a^ Crude venoms were screened for the presence of PA_366_ or PA_389_ using NMR spectroscopy.

## Supplementary Materials

The following are available online at http://www.mdpi.com/2072-6651/9/11/346/s1. **Table S1**: Crude spider venoms tested for cytotoxicity against MCF-7 cells.
