# Supplementary material for: The Aromatic Head Group of Spider Toxin Polyamines Influences Toxicity to Cancer Cells
Source: Toxins (Basel). 2017 Oct 27;9(11):346. doi: 10.3390/toxins9110346 (PMC5705961; doi:10.3390/toxins9110346)
Supplement: Supplementary file 1 [file toxins-09-00346-s001.pdf]

Supplementary Materials: The Aromatic Head Group of Spider Toxin Polyamines Influences Cellular Selectivity and Toxicity to Cancer Cells

Table 1. Crude spider venoms tested for cytotoxicity against MCF-7 cells

Species	Family:subfamily	Cytotoxic Activity*
Acanthoscurria geniculata	Theraphosidae	Yes
Aphonopelma chalcodes	Theraphosidae	Yes
Aphonopelma seemanni	Theraphosidae	No
Atrax robustus	Hexathelidae	Yes
Ceratogyrus darlingi	Theraphosidae	Yes
Chilobrachys "penang"	Theraphosidae	Yes
Chilobrachys guangxiensis	Theraphosidae	Yes
Coremiocnemis tropix	Theraphosidae	No
Grammostola porteri	Theraphosidae	No
Hadronyche cerberea	Hexathelidae	Yes
Hadronyche infensa:Newmarket	Hexathelidae	No
Hadronyche infensa:Orchid Beach	Hexathelidae	Yes
Hadronyche infensa:Toowoomba	Hexathelidae	Yes
Heteroscodra maculata	Theraphosidae	No
Hogna carolinensis	Lycosidae	Yes
Hysterocrates gigas	Theraphosidae	Yes
Lasiodora parahybana	Theraphosidae	No
Macrothele gigas	Hexathelidae	Yes
Monocentropus balfouri	Theraphosidae	No
Nhandu chromatus	Theraphosidae	Yes
Pamphobeteus antinous	Theraphosidae	No
Phlogius sp	Theraphosidae	Yes
Poecilotheria fasciata (Source 1)	Theraphosidae	No
Poecilotheria fasciata (Source 2)	Theraphosidae	No
Poecilotheria formosa	Theraphosidae	No
Poecilotheria hanumavilasumica	Theraphosidae	No
Poecilotheria miranda	Theraphosidae	No
Poecilotheria regalis	Theraphosidae	No
Poecilotheria metallica	Theraphosidae	No
Poecilotheria striata	Theraphosidae	No
Poecilotheria subfusca	Theraphosidae	No
Psalmmopoeus irminia	Theraphosidae	Yes
Pseudhapalopus spinopalpus	Theraphosidae	No
Pterinochilus meridionalis	Theraphosidae	No
Pterinochilus murinus	Theraphosidae	No
Theraphosa blondi	Theraphosidae	No
Theraphosa stirmi	Theraphosidae	Yes
Vitalius roseus	Theraphosidae	Yes

*Cytotoxic activity in MCF-7 cells was based on the crude venom having greater than 50% decrease in absorbance relative to the negative control. Species shown in red were further analysed.
